# The Activation of the Sox2 RR2 Pluripotency Transcriptional Reporter in Human Breast Cancer Cell Lines is Dynamic and Labels Cells with Higher Tumorigenic Potential

**DOI:** 10.3389/fonc.2014.00308

**Published:** 2014-11-04

**Authors:** Juan Manuel Iglesias, Olatz Leis, Estíbaliz Pérez Ruiz, Juan Gumuzio Barrie, Francisco Garcia-Garcia, Ariane Aduriz, Izaskun Beloqui, Susana Hernandez-Garcia, Maria Paz Lopez-Mato, Joaquin Dopazo, Atanasio Pandiella, Javier A. Menendez, Angel Garcia Martin

**Affiliations:** ^1^Regulation of Cell Growth Laboratory, Fundacion Inbiomed, San Sebastian, Spain; ^2^Synpromics Ltd, Edinburgh, UK; ^3^StemTek Therapeutics, Bilbao, Spain; ^4^Computational Genomics Institute, Centro de Investigación Principe Felipe (CIPF), Valencia, Spain; ^5^Functional Genomics Node, Centro de Investigación Principe Felipe (CIPF), Spanish National Institute of Bioinformatics (INB), Valencia, Spain; ^6^Instituto de Biología Molecular y Celular del Cancer, Salamanca, Spain; ^7^Centro de Investigación Biomédica en Red de Enfermedades Raras (CIBERER), Valencia, Spain; ^8^Translational Research Laboratory, Catalan Institute of Oncology (ICO), Girona, Spain; ^9^Girona Biomedical Research Institute (IDIBGi), Girona, Spain

**Keywords:** Sox2, breast cancer stem cell, pluripotency, reporter, EMT

## Abstract

The striking similarity displayed at the mechanistic level between tumorigenesis and the generation of induced pluripotent stem cells and the fact that genes and pathways relevant for embryonic development are reactivated during tumor progression highlights the link between pluripotency and cancer. Based on these observations, we tested whether it is possible to use a pluripotency-associated transcriptional reporter, whose activation is driven by the SRR2 enhancer from the Sox2 gene promoter (named S4+ reporter), to isolate cancer stem cells (CSCs) from breast cancer cell lines. The S4+ pluripotency transcriptional reporter allows the isolation of cells with enhanced tumorigenic potential and its activation was switched on and off in the cell lines studied, reflecting a plastic cellular process. Microarray analysis comparing the populations in which the reporter construct is active versus inactive showed that positive cells expressed higher mRNA levels of cytokines (IL-8, IL-6, TNF) and genes (such as ATF3, SNAI2, and KLF6) previously related with the CSC phenotype in breast cancer.

## Introduction

Cancer stem cells (CSCs) play a central role in tumor progression and recurrence, but our knowledge of their biology and origin is still limited. The lack of good CSC markers in solid tumors could explain our limited understanding of its biology and hampers the development of more efficient chemotherapy treatments. In breast cancer, fluorescent substrates (like Aldefluor), DNA dyes (such as Hoechst 33342 or Rhodamine 123 for the isolation of the side population) or different combinations of surface markers (CD24, CD44, CD133, CD49f, CD29, CD90, CD14) can be used to isolate little overlapping cell populations displaying enhanced tumor-initiating potential. To better understand the origin and dynamics of breast CSCs and to be able to use this knowledge to develop novel therapeutic approaches, new isolation methods and/or more specific combinations of markers are needed.

Cancer and developmental biology scientists realized over a century ago that genes and pathways relevant to cancer overlap with fetal development as reflected in the reactivation of embryonic genes during tumor progression. Consequently, the question was raised of whether tumors could arise from transformation of tissue stem cells or “retro-differentiation” of more differentiated cells (1). Nearly 40 years latter, these ideas and questions are still hot spots in cancer research. The “retro-differentiation” concept can be now translated as cellular plasticity, a process by which non-stem differentiated cells can spontaneously acquire stem cell-like characteristics (2). This phenomenon has important implications for cancer therapy and a big impact on our current view of the CSC hypothesis. The CSC model holds that tumors are organized in a cellular hierarchy in which CSCs are the only cells with unlimited proliferation potential and responsible for tumor growth and propagation. Originally, the CSC hypothesis was a linear model with the CSC on the top of the hierarchy and the more differentiated cells on the bottom, but the concept of cellular plasticity and experimental observations are challenging this model (3).

It is striking that the similarity observed at the mechanistic level between tumorigenesis and the generation of induced pluripotent stem (iPS) cells from fibroblasts as described by Takahashi and Yamanaka (4). The production of these iPS cells required the over-expression of four transcription factors, Oct4, Sox2, Klf4, and c-Myc, although Klf4 and c-Myc can be replaced by Lin28 and Nanog (5) and may even be dispensable. The efficiency of this reprograming process is extremely low and remains so far an *in vitro* phenomenon since there is no evidence that it can naturally occur *in vivo*. The mechanisms underlying the reprogramming process are not well understood yet; however, the three main transcription factors Oct4, Sox2, and Nanog, called master regulators of pluripotency, have proved responsible for maintaining the undifferentiated state (6, 7). Recently, the processes of reprograming and tumorigenesis have been linked as the p53 tumor suppressor, one of the main regulators of oncogenic transformation, controls the induction of pluripotency (8–10).

Both processes, reprograming and transformation, need the expression or activation of oncogenes, inactivation of tumor suppressor genes, overriding the senescence and apoptotic barriers and both processes also involve epigenetic changes and a metabolic switch toward a glycolytic metabolism (11, 12). The work from Illmensee and Mintz (13) in the mid 70s strengthens the bonds between pluripotency and cancer. They demonstrated that teratocarcinoma cells are developmentally pluripotent since single teratocarcinoma cells injected into mouse blastocysts can differentiate into many developmentally unrelated tissues. In recent years, the work from Gill Smith’s group has shown that breast CSCs are at least multipotent. Their work clearly shows that CSCs when placed in the right microenvironment can behave as phenotypically normal and can contribute to all cell types within the mammary gland epithelium (14, 15). Furthermore, it has been shown that breast CSCs have the ability to differentiate not only in epithelial but also in the endothelial lineage (16). This ability of CSCs to differentiate into unrelated cell types is also supported by the fact that glioblastoma stem/progenitor cells can differentiate into endothelial cells contributing to the vascularization of the tumor and hence to tumor progression (17).

Sox2 is a good example of a gene involved in embryonic development whose expression is reactivated during tumor generation, as Sox2 is critical to maintain the pluripotent phenotype in embryonic stem cells (ESCs) (18) and its expression is reactivated during tumor progression (19–22). Furthermore, Sox2 is part of the original Yamanaka cocktail of transcription factors necessary to reprogram somatic adult cells into iPS cells. These observations, together with the lack of reliable surface markers to isolate breast CSCs, drove us to test whether a pluripotency transcriptional GFP reporter based on the SRR2 enhancer from the Sox2 gene, developed to isolate IPS cells (23), can be used to isolate cells with cancer stem-like properties from breast cancer cell lines (24, 25). Our results showed that the activation of this transcriptional GFP reporter in breast cancer cell lines is dynamic and identifies a subpopulation of cells with enhanced tumorigenic potential. Furthermore, when cultures depleted of GFP-positive cells were established and followed over time, some cells switched on the reporter and after a while GFP-negative and GFP-positive populations reached a steady state. Interestingly, the cells in which the reporter is active display higher mRNA levels of IL6, IL8, TNF, ATF3, KLF6, or SNAI2, genes previously related with the CSC-like phenotype and cellular plasticity in breast tumors.

## Materials and Methods

### Cell lines and culture conditions

MCF7 and MDA-MB-231 breast carcinoma cell lines were obtained directly from ATCC (Manasses, VA, USA) and were grown in DMEM (Gibco, Carlsbad, CA, USA) supplemented with 10% fetal bovine serum (Sigma, St. Louis, MO, USA) and 1% Penicillin/Streptomycin (Sigma, St. Louis, MO, USA). MDA-MB-436 cell line was a kind gift from T. Stein (University of Glasgow, UK, previously obtained from ATCC, Manassas, VA, USA) and was grown in DMEM (Gibco, Carlsbad, CA, USA) supplemented with 10% fetal bovine serum (Sigma, St. Louis, MO, USA), 20 ng/ml Insulin (Sigma, St. Louis, MO, USA) and 1% penicillin/streptomycin (Sigma, St. Louis, MO, USA). All the cell lines were kept at 37°C in a 5% CO_2_ incubator.

### Mouse xenograft assays

Female 6-week-old athymic nude mice (Balb/c Nu/Nu) were purchased from Charles River, and were housed in specifically designed pathogen-free isolation animal facility. All animal procedures were performed in accordance with institutional animal care and use guidelines and approved by the IRB. GFP^High^ and GFP^Low^ MCF7 cells were resuspended in 200 μl of PBS with matrigel and subcutaneously inoculated in left and right caudal mammary fat pads. In all, 2.5 × 10^6^; 0.5 × 10^6^, and 0.25 × 10^6^ GFP^High^ MCF7cells were inoculated in the right mammary fat pad, with their respective GFP^Low^ MCF7 controls in the left mammary fat pad. Mice were weighed and the inoculation sites were inspected by palpation at weekly intervals. When tumors become detectable manually, the growth rates were determined by weekly measurement of two diameters of the tumor with a Vernier caliper. The tumor volume was estimated as the volume of an ellipse using the following formula: *V* = 4/3 × (*a*/2) × (*b*/2)2, where “*a*” and “*b*” correspond to the longest and shortest diameter, respectively. Animals were euthanized when their tumors were harvested.

### Flow cytometry and microscopy

Cells were harvested by trypsinization, trypsin was inactivated with regular medium, and DNAse I was added at a final concentration of 0.2 mg/ml, cell suspensions were incubated at 37°C for another 10 min and spun down, and finally cell pellets were resuspended in a suitable volume of sorting buffer (PBS w/o Ca and Mg, 1% BSA, 5 mM EDTA). TO-PRO-3 (Molecular Probes, Life Technologies) was added as dead cell indicator and BD FACSAria or BD FACSCanto machines were used for sorting and analysis experiments following the gating strategy depicted on the Figure S2 in Supplementary Material. When tracking the changes in the percentage of GFP^High^ cells over time the FACSCanto was calibrated prior to the analysis using the Spherotech Rainbow beads (Spherotech Inc., Lake Forest, IL, USA) to ensure consistent signals over the course of the experiment and verify proper function of the machine.

### Microarray analysis

Total RNA from freshly sorted MCF7S4+ GFP^High^ and GFP^Low^ cells was prepared using TRIzol (Life Technologies) and whole genome gene expression analysis was performed using the HumanHT-12 v4 Expression BeadChip platform (Illumina) containing 47323 probes per chip. Data were standardized using background correction and quantile normalization (26). Differential gene expression was carried out using the limma (27) package from Biocondui (http://www.bioconductor.org/). We performed a statistical test for each probe according to Benjamini and Hochberg (28) methodology. Gene set analysis was carried out for the Gene Ontology terms using FatiScan (29) in Babelomics (30) (http://babelomics.bioinfo.cipf.es/). This is a web-based program for the functional interpretation of large-scale experiments. The test aims to directly test the behavior of blocks of functionally related genes, instead of focusing on single genes. This tool detects significantly up- or downregulated blocks of functionally related genes in lists of genes ordered by differential expression. FatiScan returns adjusted *p*-values based on false discovery rate (FDR) method (28, 31). Significant GO terms were represented by directed acyclic graphs from Blast2GO (32). GO annotation for the genes in the microarray where taken from Ensembl 56 release (http://www.ensembl.org).

### Lentiviral gene transfer

Lentiviral particles encoding the pluripotency transcriptional reporter pL-SIN-EOS-S(4+) EGFP (23) were produced in-house at the Viral Vectors Core Unit. Cell lines were plated the day before the infection in six-well plates at a cell density of 0.25 × 10^6^ cells per well and exposed to the lentiviral particles at a MOI 2.5 in serum free medium for 6 h, cells were washed twice with serum free medium and kept in regular medium thereafter.

## Results

### The S4+ transcriptional reporter is active in breast cancer cell lines

**Figure 1:**
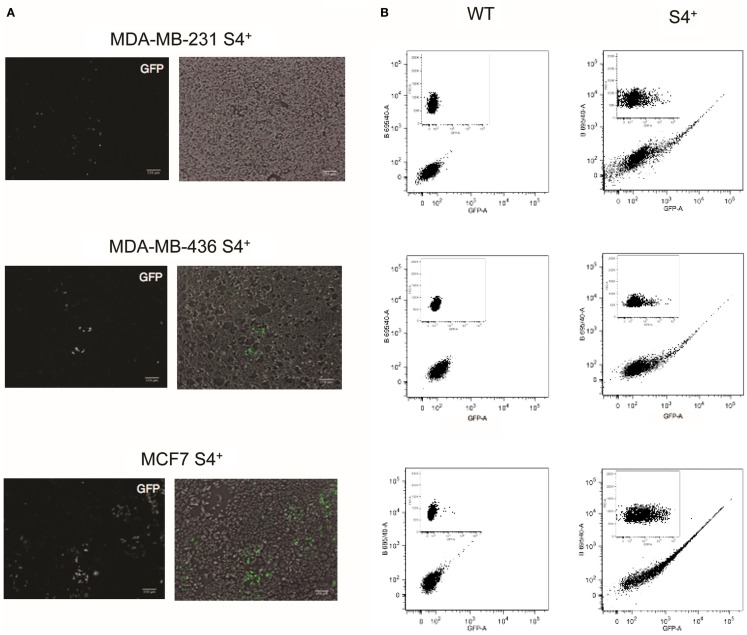
**S4+ pluripotency transcriptional reporter is active in breast cancer cell lines**. **(A)** On the left, fluorescence images of MDA-MB-231, MDA-MB-436, and MCF7 cell lines infected with the S4+ reporter to detect GFP expression. On the right are shown the fluorescence image (in green) merge with the bright-field image. **(B)** FACS plots of the wild-type cell lines MDA-MB-231, MDA-MB-436, and MCF7 and the S4+ derivatives infected with the S4+ reporter. On the bigger plot, GFP fluorescence is displayed on the *X*-axis and the fluorescence collected through the 695/40 filter on the *Y*-axis. On the inset, GFP fluorescence is displayed on the *X*-axis and the forward scattering on the *Y*-axis.

The pL-SIN-EOS-S(4+) EGFP pluripotency transcriptional reporter (from now on S4+ reporter) was described by Hotta et al. (23) as a tool to isolate human iPS cells. The backbone of this reporter is based on the EOS lentiviral system and the synthetic promoter controlling the expression of the EGFP reporter is made of a minimal promoter sequence derived from the LTR promoter from an early transposon (ETn) and four tandem repeats of the SRR2 enhancer sequence from the Sox2 gene (Figure S1 in Supplementary Material). To test whether this pluripotency reporter is active in breast cancer cell lines, cell lines MCF-7S4+ (representing the most common luminal breast cancer type), MDA-MB-231S4+ (as example of mesenchymal-like breast carcinoma), and MDA-MB-436S4+ (representing BRCA1 deficient breast cancer) were generated from parental cell lines through lentiviral gene transfer of the pL-SIN-EOS-S(4+) EGFP transcriptional reporter. The activation of the transcriptional reporter was analyzed by fluorescence microscopy to detect GFP expression. The three S4+ derivative cell lines expressed different levels of GFP in individual cells as shown in Figure 1. To quantify the number of cells expressing GFP and its expression levels, FACS analysis was performed. As shown in Figure 1, most of the GFP-positive cells expressed low levels of GFP with just a few cells expressing high levels of GFP in the three cell lines.

### Cells in which S4+ reporter is active are more tumorigenic

One of the first questions we made after we found out that the S4+ reporter is active in a small population of cells was if there is any difference in tumorigenic potential between the GFP+ and GFP− cells. Before we could address this question, we performed a calibration experiment to find out the minimum GFP levels detected on the FACS machine that can be detected by the naked eye on the microscope to help us decide which populations to select for the assay. MCF7S4+ cells were used to establish regions of fluorescence intensity (termed P2-P11) so cells falling in gates P10, P11, and P3 were GFP fluorescent when examined under the fluorescent microscope, as shown in Figure S2 in Supplementary Material, thus we decided to use cells in gate P3 as GFP^High^ for further studies. We decided to select the gate P4 as GFP^Low^ and not one of the gates on its right because in the latter ones there is a potential mixture of cells in which the reporter is inactive and cells lacking any viral integration. As control, expression of Sox2 was checked through RT-PCR, showing increased expression of Sox2 in GFP^High^ cells compared to GFP^Low^ cells, as expected.

**Figure 2:**
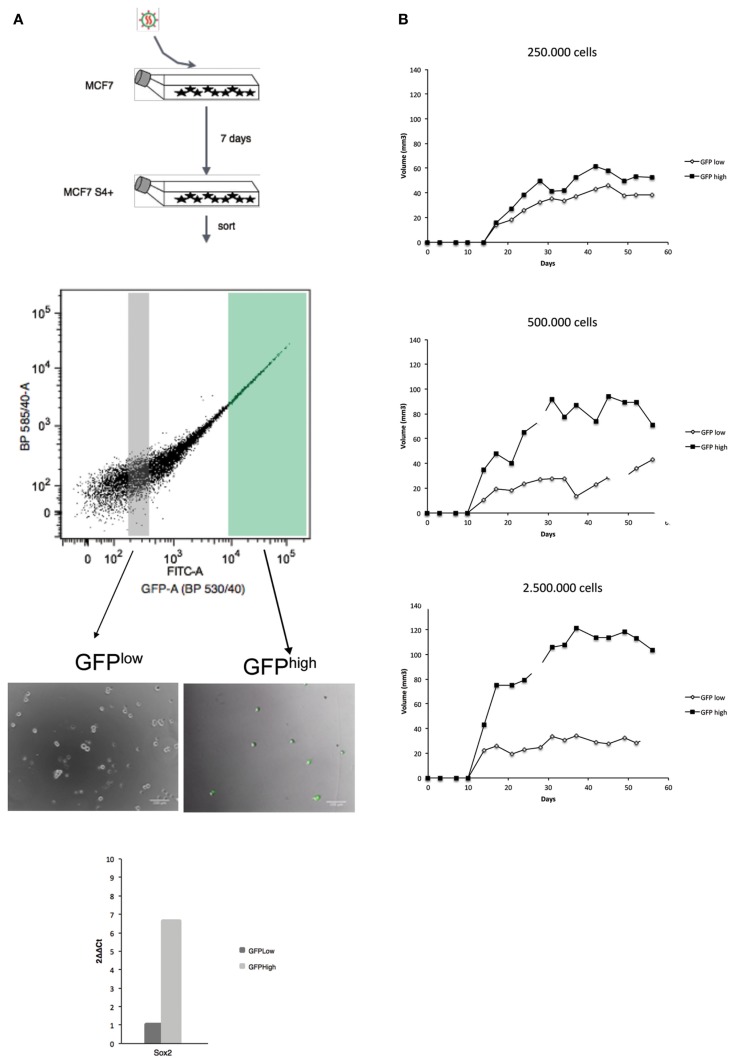
**Cells in which the S4+ reporter is active show higher tumorigenic potential in NOD/SCID mice**. The outline of the experiment is shown on the left **(A)** and the outcome on the right **(B)**. **(A)** MCF7 cells were infected with the lentiviral reporter vector, 7 days later GFP^High^ and GFP^Low^ populations were sorted, GFP expression verified by fluorescent microscopy and SOX2 mRNA differential expression assessed by qPCR. **(B)** The GFP^High^ and GFP^Low^ cells were culture for 2 days and subcutaneously injected into the left (GFP^Low^) or right (GFP^High^) fat pads of 6-week-old female nude mice and tumor growth was monitored weekly. In these experiments, three animals per condition were used and the standard deviation is plotted for each time point.

On the basis of these results, we tested if there is any difference in tumorigenic potential between GFP^High^ and GFP^Low^ cells in the MCF7S4+ cell line. GFP^High^ and GFP^Low^ populations were then FACS sorted, injected subcutaneously in each flank of nude female mice, and tumor growth was monitored for 8 weeks. As shown in Figure 2, tumors coming from GFP^High^ cells grew out first and faster than tumors initiated by GFP^Low^ cells, and this difference is more evident when higher numbers of cells are injected. We used MCF7S4+ cells as model for tumorigenesis in xenograft experiments instead of a mesenchymal-like model of breast cancer (such as MDA-MB-231S4+) because mesenchymal-like breast carcinoma cells are very invasive and spread rapidly when xenografted to immunocompromised mice, making this model unfeasible to compare direct tumorigenicity.

### The S4+ transcriptional reporter is dynamic in breast cancer cell lines

**Figure 3:**
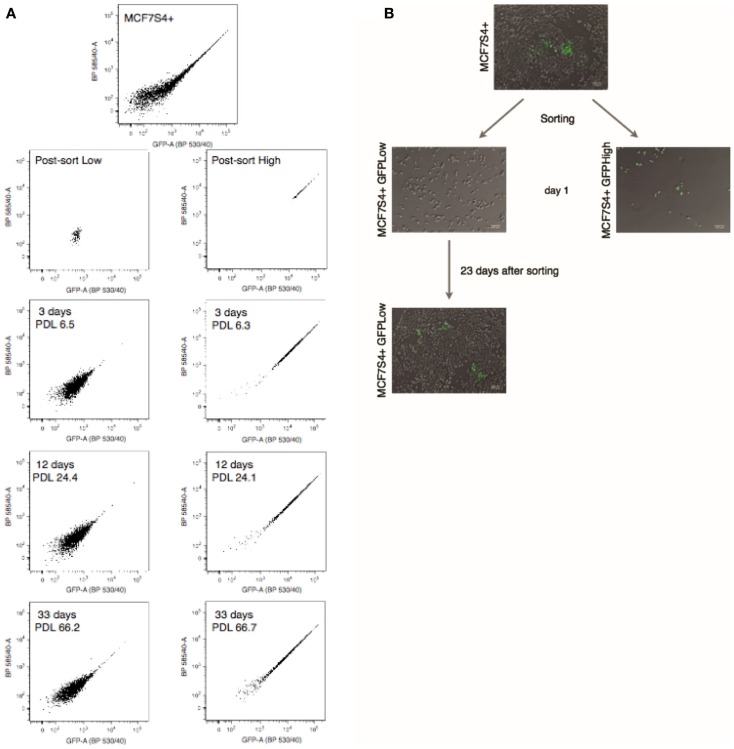
**The S4+ reporter is dynamic in MCF7 cells**. **(A)** FACS plots of the parental MCF7S4+ cell line, the GFP^High^ and GFP^Low^ populations just after sorting, and after 3, 12, and 33 days in culture. **(B)** Typical micrographs of cell cultures at indicated time points. The GFP^High^ and GFP^Low^ populations were cultured on its own and changes in fluorescence were monitored by FACS at the indicated time points. Population doublings after sorting are also indicated for each time point.

Hotta et al. have shown that the S4+ reporter is dynamic; it is off in non-pluripotent cells, such as fibroblasts, turns on in iPS cells, and turns off again when the iPS cells are induced to differentiate into any lineage. To test whether the reporter is also dynamic in breast cancer cell lines, GFP^High^ and GFP^Low^ populations where sorted, placed in culture and changes in fluorescence where monitored by FACS analysis at each passage. When GFP^Low^ cells are placed in culture, the heterogeneity of the parental cell line is restored after just few days in culture (Figure 3), the same is true for the GFP^High^ population. The S4+ transcriptional reporter is also dynamic in MDA-MB-231 and MDA-MB-436 breast carcinoma cell lines, as shown in Figure S3 in Supplementary Material.

To confirm that the transcriptional reporters are dynamic and the restoration of the heterogeneity observed in the original cell line is not due to contamination during the sorting process, a clonogenic assay was set up using the MCF7 S4+ cell line. To carry on this assay, individual GFP^High^ or GFP^Low^ cells were FACS sorted into each well of a 96-well plate, each well was checked for the presence of an individual cell at the microscope, and after 3 weeks, the colonies were scored for the presence of mixed-colonies with GFP^+^ or GFP^−^ cells by fluorescence microscopy (experimental outline depicted in Figure S4 in Supplementary Material). This assay shows that the frequency of firing is much lower than the frequency of extinction of GFP as it would be expected if GFP labels CSCs and the dynamic activity of the transcriptional reporter reflects cellular plasticity (Table 1).

**Table 1:** **Frequency of reporter activation and inactivation through single cell plating**.

	Number of positions	GFP^low^	GFP^high^
Day 1	Analyzed	288	288
	Containing 1 cell	212	256
After 3 weeks	With colonies	98 (46.2%)	144 (56.2%)
	With colonies made of GFP+ and GFP−cells	9 (9.1%)	76 (52.7%)

In these series of experiments, we observed that when MCF7S4+ GFP^Low^ cells are placed in culture they switch on the S4+ reporter, and after a few passages, the culture reached a steady state in which the percentage of GFP^High^ cells stays around 0.1–0.3%.

### Identification of genes differentially expressed among GFP^High^ and GFP^Low^ populations

**Figure 4:**
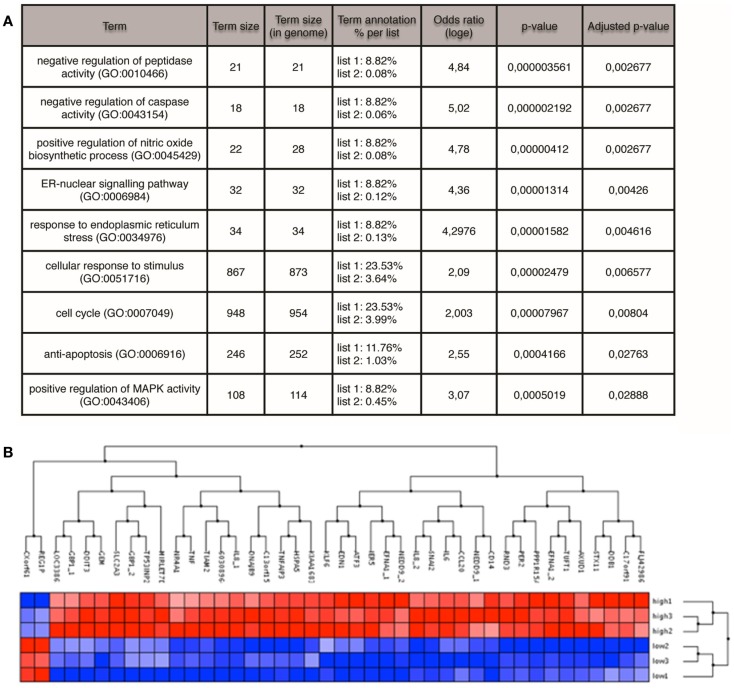
**Microarray profiling of GFP^High^ versus GFP^Low^ MCF7 cells**. **(A)** GO terms enriched in the GFP^High^ population identified by FatiScan enrichment analysis. **(B)** Genes (columns) differentially expressed between GFP^High^ and GFP^Low^ cells (rows). Red color denotes high expression, blue low expression.

GFP^High^ and GFP^Low^ populations from the MCF7S4+ cell line were isolated by FACS and total RNA was prepared to perform microarray analysis on the Illumina HumanHT-12_V4 BeadChip platform. Results were normalized and analyzed using Bioconductor and Babelomics, showing that 42 genes were found differentially expressed between the two populations with an adj. *p*-value < 0.1, with 40 of those genes showing higher expression in the GFP^High^ population (Figure 4). Among the genes upregulated in GFP^High^ cells are cytokines (IL-6, IL-8, or TNF) and transcription factors (KLF6, ATF3, SNAI2) that have been previously related with cancer stemness, cellular plasticity, or both. GO analysis showed enrichment in genes related to anti-apoptosis (GO:0006916) and positive regulation of nitric oxide biosynthetic process (GO:0045429) among others (Figure 4). Interestingly, increased nitric oxide synthase expression in estrogen receptor-negative breast cancer patients predicts poor survival (33) and the enrichment in anti-apoptotic genes can contribute the intrinsic chemoresistance characteristic of BCSCs (34). Further experimentation will be needed to validate the links between these processes, specially inflammation, and CSC induction.

## Discussion

Different combinations of surface markers have been described to isolate CSCs, but it is striking that little overlap has been found between CSC markers reported in different tumor types (35). Prominin (CD133) is a good marker for brain and colon CSCs, but has never been successfully used for isolating breast CSCs. Even within breast tumors, the accepted combination of surface markers CD44/CD24 shows differences among different subtypes, being the CD44+/CD24− phenotype common in the basal subtype, specially in BRCA1 hereditary tumors, but surprisingly scarce in HER2-positive tumors (36). In this work, we utilize transcription programs unique in stem cells as a new method to report the activity of CSCs.

The work from Illmensee and Mintz (13) linked teratocarcinoma cells to pluripotency. Regulatory networks orchestrated by key transcription factors like SOX2, OCT4, and NANOG have been proposed to play an important role maintaining ESC identity (6, 7). Interestingly, mRNA profiling studies suggest that ESC and CSCs share common transcriptional programs (37). Furthermore, transcriptional reporters containing regulatory regions derived from those genes had previously been successfully used to isolate CSCs (38–40). These observations and the striking similarity observed at the mechanistic level between tumorigenesis and the generation of iPS cells prompted us to test whether a pluripotency transcriptional reporter developed to isolate human iPS cells could be also used in the isolation of CSCs. The pluripotency transcriptional reporter selected to test our hypothesis was the pL-SIN-EOS-S(4+) EGFP (in short, S4+ reporter) (23). The synthetic promoter driving the expression of GFP is made of four tandem repeats of the SRR2 enhancer from the Sox2 gene plus the LTR from the mouse ETn. These transposons are only active during early mouse embryogenesis in ESCs and embryonic carcinoma (EC) cells. This configuration using a minimal promoter only active in ESCs and ECs and an enhancer sequence derived from the regulatory region of one of the key transcription factor for the maintenance of the ESC identity may provide a more specific way of isolating CSCs and may allow the isolation of CSCs from different tumor sources. Previous work from our laboratory (19) and others (41, 42) had shown that Sox2 gene is activated in the early phases of breast tumor development and necessary for tumorigenicity of MCF7 cells; therefore, a reporter based on Sox2 promoter elements seemed appropriate.

After breast cancer cell lines are infected with the S4+ lentiviral transcriptional reporter, only a small fraction of cells switched on the expression of the GFP repoter, when inspected under the fluorescence microscope (GFP^High^). Interestingly, the GFP^High^ cells showed enhanced tumorigenicity when injected into immunocompromised female mice.

The S4+ pluripotency transcriptional reporter is active in iPS and ES cells, but it is turned off when iPS cells are induced to differentiate (23). Here, we show for the first time that this reporter is also dynamic in breast cancer cell lines, as cell cultures depleted of GFP^High^ cells show spontaneous conversion of GFP^Low^ cells (these are the cells GFP-negative at the microscope, but with background GFP expression – measured through flow cytometry, demonstrating effective viral integration but not transcriptional activation of the reporter) into GFP^High^ and after a few passages the culture reached a steady state, similar to the parental culture. This phenomenon is reminiscent of cellular plasticity as described by Chaffer et al. (2), as the spontaneous conversion to a stem-like state of non-stem cells. The reverse is also true, when S4+ cell lines were depleted of GFP^Low^ cells, some GFP^High^ cells switched off the expression of the reporter becoming GFP^Low^, this might be equivalent to a differentiation process. In the breast cancer cell lines tested, the percentage of cells in which the reporter is active, ranges between 0.4 and 8% after transduction, this percentage fell below 1% when GFP^Low^ cultures were established and allowed to reach its steady state. These discrepancies in the percentage of fluorescent cells may be due to differences in the transduction efficiency and non-specific activation of the reporter due to positional effects after the lentiviral integration in the genome. Working with steady-state cultures derived from GFP^Low^ cells reduces the unspecific activation of the reporter due to positional effects. We used cell lines representing the main subtypes of breast cancer to prevent cell line bias: MCF7 as luminal ER-dependent breast cancer, MDA-MB-231 as mesenchymal-like basal breast carcinoma, and MDA-MB-436 as a model of hereditary BRCA1-deficient breast cancer. Similar results were obtained from all cell lines. Expression of the reporter gene did not alter phenotypic features of the cell lines used, such as ER expression in MCF7 cells (data not shown). Moreover, we recently published (43) a link between E2/ERa signaling in breast cancer and pluripotency-like reporgramming, pointing to a mechanism where SOX2 can promote non-genomic E2 signaling that leads to nuclear phospho-Ser118-ERa, which exacerbates genomic ER signaling in response to E2. Since E2 stimulation has been recently shown to enhance breast tumor-initiating cell survival (through downregulation of miR-140), which targets SOX2, this suggests a bidirectional cross-talk interaction to regulate breast cancer activity.

In order to understand the mechanisms governing the interconversion of reporter positive and negative cells, transcriptional profiling was carried out. Comparison of GFP^High^ versus GFP^Low^ populations showed many genes previously related with CSC homeostasis upregulated in the GFP^High^ population, where the reporter is active. The cytokines IL-6 and IL-8 are among the upregulated genes in GFP^High^ cells. IL-6 secretion has been reported to modulate the inducible formation of breast CSCs and their dynamic equilibrium with non-stem cancer cells (44) and recombinant IL-8 increased mammosphere formation and the ALDEFLUOR-positive population in breast cancer cell lines (25). The transcription factor ATF3 acts as an oncogene in mouse mammary gland (45) and enhances TGFβ signaling and CSC features in breast cancer cell lines (46). We found also genes related with epithelial-to-mesenchymal transition (EMT) upregulated in the GFP^High^ population, such as SLUG or NEDD9. Recent studies suggest a link between EMT and acquisition of stem cell properties (47, 48) where Slug co-operates with a Sox family member (Sox9) in the reprogramming of differentiated luminal epithelial cells to a stem-like state in the mouse mammary gland and co-expression of both transcription factors in breast cancer is associated with patient survival (49). NEDD9 acts as a positive regulator of EMT in breast cancer cell lines (50), and it is also involved in mammary gland tumorigenesis (51, 52).

These data are compatible with a model of inducible formation of CSCs and their dynamic equilibrium with non-stem cancer cells. Further experimentation is needed to fully understand the molecular determinants controlling this process, which may have significant impact in our understanding of tumor generation and progression, and therefore opening new possibilities for therapeutic intervention. In this work, we demonstrate the use of a pluripotency related promoter as a tool to track CSC phenotype acquisition in breast cancer, suitable for novel drug discovery targeting the CSC compartment.

## Conflict of Interest Statement

Juan Manuel Iglesias is an employee of the private commercial company Synpromics Ltd. Angel Garcia Martin and Olatz Leis own stock of the private commercial company StemTek Therapeutics. Juan Gumuzio Barrie is an employee of the private commercial company StemTek Therapeutics. The other co-authors declare that the research was conducted in the absence of any commercial or financial relationships that could be construed as a potential conflict of interest.

## Supplementary Material

The Supplementary Material for this article can be found online at http://www.frontiersin.org/Journal/10.3389/fonc.2014.00308/abstract
